# To what extent could cardiovascular diseases be reduced if Germany applied fiscal policies to increase fruit and vegetable consumption? A quantitative health impact assessment

**DOI:** 10.1017/S1368980020000634

**Published:** 2021-06

**Authors:** Johanna-Katharina Schönbach, Stefan K Lhachimi

**Affiliations:** 1 Institute of Public Health and Nursing Research, University of Bremen, Bremen, Germany; 2 Research Group for Evidence Based Public Health, Leibniz Institute for Prevention Research and Epidemiology – BIPS, Bremen, Germany

**Keywords:** Health impact assessment, Modelling, Fruits, Vegetables, Policy

## Abstract

**Objective:**

This study aimed to dynamically model and quantify expected health effects of four scenarios: (i) a reference scenario with an unchanged fruit and vegetable intake, (ii) the removal of value-added tax (VAT) on fruits and vegetables, (iii) the implementation of a 20 % subsidy on fruits and vegetables and (iv) a guideline scenario with a population-wide fruit and vegetable intake of five portions per day.

**Design:**

Baseline fruit and vegetable intake data was derived from the GEDA 2012 study. We used price elasticities for Germany to calculate the change in fruit and vegetable consumption under the zero VAT and the 20 % subsidy scenario. All scenarios were modelled over a 10-year projection period using DYNAMO-HIA.

**Setting:**

Germany.

**Participants:**

A projected real-life population.

**Results:**

Cumulated over the 10-year projection period, an estimated 4450 incident ischaemic heart disease (IHD) cases, 7010 stroke cases and 13 960 deaths would be prevented under the zero VAT scenario. Under the 20 % subsidy scenario, 17 990 incident IHD cases, 27 390 stroke cases and 54 880 deaths would be averted. Although this corresponds to only a fraction of the incidents that would occur under the reference scenario, the averted cases translate to 2 % (for the zero VAT scenario) and 9 % (for the 20 % subsidy scenario) of IHD, stroke and death cases that would be prevented if the whole population consumed the recommended five portions of fruits and vegetables per day.

**Conclusions:**

Fiscal policies on fruits and vegetables provide a non-negligible step towards the removal of the health burden induced by low fruit and vegetable intake.

Fruit and vegetable intake reduces the risk of ischaemic heart disease (IHD) and stroke^(1)^. For a healthy diet, the WHO recommends consuming 400 g (i.e. five 80-g portions) per day^(2)^. Nevertheless, Eurostat data shows that only 14·3 % of the EU-28 population reach this recommendation^(3)^.

According to the German nutrition report from 2019, 29 % of the population do not even consume fruits and vegetables on a daily basis^(4)^. The Federal Ministry of Food and Agriculture that published the report generally emphasises an individual’s self-responsibility to eat healthily. Its National Action Plan suggests a range of programmes and projects that encourage the dissemination of information as well as the creation of structures for healthy lifestyles in schools, workplaces and the community, whereas fiscal policies are not included^(5)^.

Notwithstanding, measures targeting informed choice (individual-based education, public information campaigns) have shown only weak effectiveness to improve diet^(6–8)^. Additionally, individual-based information and education appear to increase socioeconomic inequalities in diet^(9)^, because they build on individuals’ resources^(10)^. In contrast, fiscal measures have shown stronger evidence for success^(6,8)^ and may decrease socioeconomic inequalities^(9)^.

The use of economic tools, such as targeted subsidies and taxes, has been recommended by the WHO in its European Food and Nutrition Action Plan 2015–20^(11)^. In fact, experimental and observational studies have already found that subsidies on healthier foods, such as fruits and vegetables, modify dietary behaviour in the desired direction^(12–15)^. However, the assessment of expected health impacts from a potential fruit and vegetable price change has relied on simulation studies so far. Health effects have previously been modelled in the form of a 1 %^(16)^, 10 % and a 30 % subsidy on fruits and vegetables in the United States^(17–19)^, a 20 % subsidy in New Zealand^(20)^, as well as a subsidy of $0·14 per 100 g on fruits and vegetables in Australia^(21)^. With regard to value-added tax (VAT) changes, it was estimated that halving the VAT rate on fruits and vegetables (from 25 to 12·5 %) would save 367·2 disability-adjusted life years in the Danish population^(22)^. Similarly, a 3·4 % reduction in VAT would avoid 363 deaths and save 5024 life-years in the French population^(23)^.

For Germany, it had been estimated that reducing the German VAT rate for fruits and vegetables from the current 7 to 0 % and increasing the VAT rate of unhealthy foods from 7 to 19 % according to a traffic light system could decrease obesity by 3 % in females and 8 % in males^(24,25)^.

The aim of this study was to further quantify the prevention potential of fiscal policies on fruits and vegetables – in this case on the health outcomes of IHD, stroke and deaths. We therefore project two specific fruit and vegetable subsidies over a period of 10 years, in comparison to a reference scenario (in which intake remains unchanged) and a guideline scenario (in which everyone in the population consumes the recommended five portions of fruits and vegetables per day).

## Methods

### Scenarios

We ran four scenarios: (i) In the reference scenario, we assumed the fruit and vegetable intake would remain unchanged. (ii) The zero VAT scenario hypothesised that the VAT for fruits and vegetables would be reduced from the current 7 to 0 %, translating into a price decrease of 6·54 %. (iii) In the 20 % subsidy scenario, we presumed a subsidy on fruits and vegetables of 20 %, as has previously been the subject of research for New Zealand^(20)^. (iv) Under the guideline scenario, the whole population is supposed to consume the recommended five portions of fruits and vegetables per day^(2)^.

### Price elasticities

Price elasticities express the percentage change in demand in response to a 1 % change in price^(26)^ and have likewise been applied in similar studies^(19,21,23,27)^. Thus, we used Germany-specific own-price elasticities taken from literature^(28)^ to estimate what impact the respective price changes induced by the zero VAT scenario and the 20 % subsidy scenario would have on fruit and vegetable demand. According to these price elasticities, the zero VAT scenario with a fruit and vegetable price reduction of 6·54 % would increase fruit intake by 5·2 % and vegetable intake by 3·6 % (see Table 1). The 20 % subsidy scenario with its 20 % price decrease would lead to a 16 % increase in fruit intake and a 11 % increase in vegetable intake (see Table 1).

**Table 1:** Uncompensated (Marshallian) own-price elasticities from Thiele 2008^(28)^

Item	Elasticity	Illustration: Fruit and vegetable demand change after…	
		…a 6·54 % price decrease (zero VAT scenario)	…a 20 % price decrease (20 % subsidy scenario)
Fruits	–0·80	+5·2 %	+16·0 %
Vegetables	–0·55	+3·6 %	+11·0 %

### Fruit and vegetable intake over scenarios

For the reference scenario, we derived individual fruit as well as vegetable intake from 19 189 persons who gave complete fruit and vegetable information in the public use file of the German Health Update study 2012 (GEDA 2012)^(29)^. GEDA 2012 was carried out between March 2012 and March 2013 by the Robert Koch Institute^(30)^. In the survey, fruit and vegetable intake was assessed separately. Participants were asked whether they consumed fruits and vegetables, respectively, ‘every day’, ‘at least once per week’, ‘less than once per week’ or ‘never’. If they stated they consume fruits or vegetables every day, they were asked to report the number of daily portions. If they stated they consume the respective item at least once per week, they were asked to report the number of weekly portions, which we then converted to the number of daily portions. For fruits, the weighted mean intake was 1·17 portions per day. For vegetables, the weighted mean intake was 0·93 portion per day.

For the zero VAT and 20 % subsidy scenarios, we applied the intake change as estimated by the price elasticities to the individual fruit and vegetable intake data as observed under the reference scenario.

**Fig. 1:**
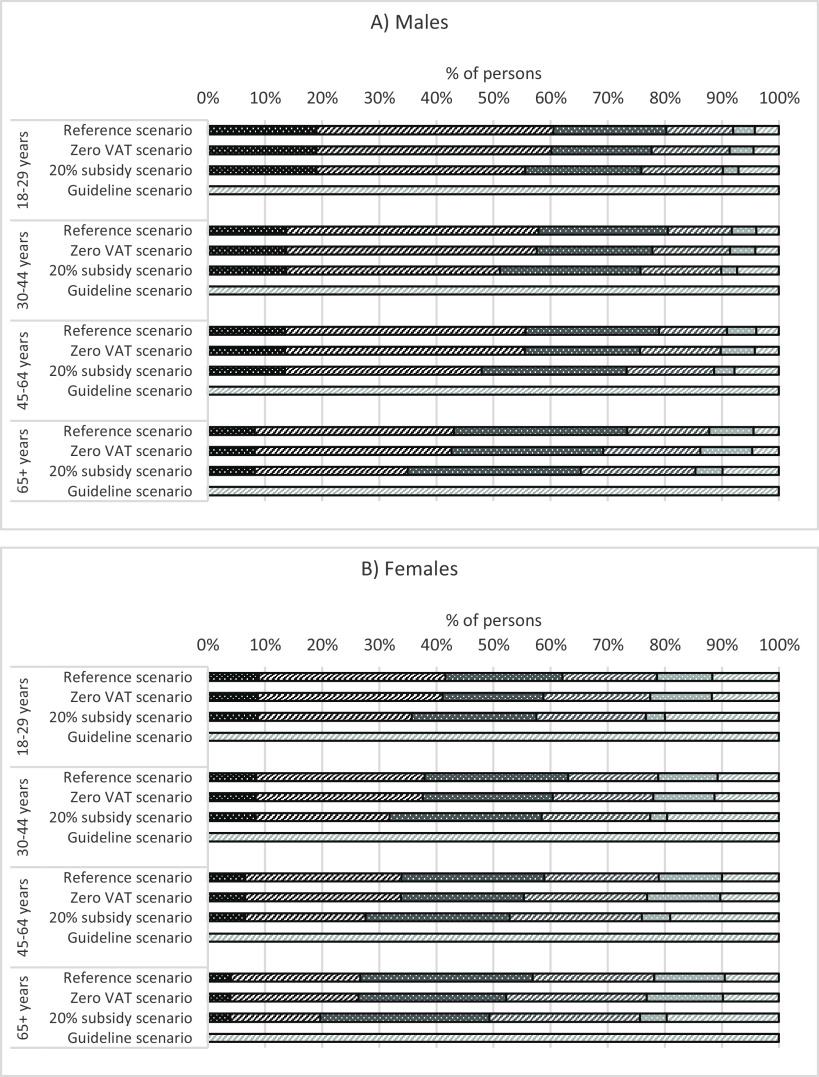
Fruit and vegetable intake over scenarios, before smoothing over age. 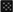, 0 to ≤0·5 portions; 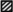, >0·5 to ≤1·5 portions; 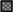, >1·5 to ≤2·5 portions; 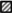, >2·5 to ≤3·5 portions; 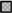, >3·5 to ≤4·5 portions; 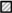, >4·5 portions

In order to obtain a combined fruit and vegetable variable per scenario, we added the portions of fruits and vegetables per person. This intake variable was then categorised into 0 to ≤0·5 portions, >0·5 to ≤1·5 portions, >1·5 to ≤2·5 portions, >2·5 to ≤3·5 portions, >3·5 to ≤4·5 portions and >4·5 portions for males and females and four age groups (18–29, 30–44, 45–64, 65+). For the guideline scenario, we assumed everyone to be in the category of >4·5 portions. The fruit and vegetable intake over all four scenarios is illustrated in Fig. 1. Finally, we smoothed the intake proportions over age using multinomial P-splines^(31,32)^.

### Relationship between fruit and vegetable intake and health outcomes

Relative risks per 200 g of fruits and vegetables per day for all-cause mortality, IHD and stroke were based on a published meta-analysis^(33)^. Our intake category-specific relative risks (>0·5 to ≤1·5 portions, >1·5 to ≤2·5 portions, >2·5 to ≤3·5 portions, >3·5 to ≤4·5 portions and >4·5 portions) are presented in Table 2.

**Table 2:** Relative risks for fruits and vegetables

Outcome	Relative risks per unit, source	Intake category-specific relative risks					
		0 to ≤0·5 portions	>0·5 to ≤1·5 portions	>1·5 to ≤2·5 portions	>2·5 to ≤3·5 portions	>3·5 to ≤4·5 portions	>4·5 portions
All-cause mortality	0·90 (0·87–0·93) per 200 g/d of fruits and vegetables; Aune *et al.* ^(33)^	1·00	0·96	0·92	0·88	0·84	0·81
IHD	0·92 (0·90–0·94) per 200 g/d of fruits and vegetables; Aune *et al.* ^(33)^	1·00	0·97	0·94	0·90	0·88	0·85
Stroke	0·84 (0·76–0·92) per 200 g/d of fruits and vegetables; Aune *et al.* ^(33)^	1·00	0·93	0·87	0·81	0·76	0·71

IHD, ischaemic heart disease.

### Population and disease data

We derived Germany’s age- and sex-specific population size, mortality, projected births, as well as prevalence, incidence and excess mortality for IHD and stroke from the DYNAMO-HIA database. The database is freely available on the project’s website (https://www.dynamo-hia.eu)^(34)^ and has been used in several studies^(35–37)^.

### Health outcome assessment: DYNAMO-HIA

The DYNAMO-HIA software tool^(34,38,39)^ was used to project a real-life population under all four scenarios through fruit and vegetable intake biographies and associated diseases. In our analysis, we ran a projection period of 10 years, and compared deaths as well as incident and prevalent IHD and stroke cases among males and females between the four scenarios. DYNAMO-HIA has previously been used to model health impacts following changes in risk factors, such as alcohol consumption^(27)^, smoking^(35,40,41)^ and second-hand smoke^(37)^, BMI^(32)^, salt intake^(36,42)^ and physical activity^(43)^.

## Results

The difference in incident and prevalent IHD and stroke cases between the reference, zero VAT, 20 % subsidy as well as the guideline scenario over the projection period is illustrated in Table 3. The difference in deaths between scenarios is illustrated in Table 4.

**Table 3:** Incident and prevalent cases of IHD and stroke

Outcome	Scenario							Prevalent cases in projection year 10					
		Number of incident cases after the first year of projection			Cumulative number of incident cases over 10-year projection period			Males		Females		Total	
		Males	Females	Total	Males	Females	Total	*n*	%	*n*	%	*n*	%
IHD	Ref	139 170	117 920	257 090	1 515 880	1 250 190	2 766 070	2 052 030	5·2	1 642 450	4·0	3 694 480	4·6
	Δ of zero VAT scenario to Ref	–260	–250	–510	–2270	–2180	–4450	–640		–660		–1300	
	Δ of 20 % subsidy scenario to Ref	–1020	–990	–2010	–9210	–8780	–17 990	–2930		–2850		–5780	
	Δ of guideline scenario to Ref	–13 590	–9490	–23 080	–126 940	–85 730	–212 670	–45 660		–28 070		–73 730	
Stroke	Ref	83 430	87 830	171 260	943 910	950 440	1 894 350	833 520	2·1	766 530	1·9	1 600 050	2·0
	Δ of zero VAT scenario to Ref	–330	–400	–730	–3260	–3750	–7010	–1620		–1790		–3410	
	Δ of 20 % subsidy scenario to Ref	–1280	–1550	–2830	–12 740	–14 650	–27 390	–6560		–7120		–13 680	
	Δ of guideline scenario to Ref	–16 180	–14 330	–30 510	–164 730	–138 620	–303 350	–86 230		–67 550		–153 780	

IHD, ischaemic heart disease; Ref, reference scenario; VAT, value-added tax.

**Table 4:** Number of deaths

Scenario	Number of deaths after the first year of projection			Cumulative number of deaths over 10-year projection period		
	Males	Females	Total	Males	Females	Total
Ref	357 040	391 020	748 060	4 130 430	4 376 070	8 506 500
Δ of zero VAT scenario to Ref	–710	–860	–1570	–6740	–7220	–13 960
Δ of 20 % subsidy scenario to Ref	–2700	–3430	–6130	–26 290	–28 590	–54 880
Δ of guideline scenario to Ref	–35 270	–32 290	–67 560	–350 450	–278 380	–628 830

Ref, reference scenario; VAT, value-added tax.

### Ischaemic heart disease, stroke and death incidents after the first year of projection

Under the reference scenario, that is, an unchanged fruit and vegetable intake, 257 090 incident IHD cases, 171 260 incident stroke cases and 748 060 deaths would occur by the end of the first year of projection. In comparison to the reference scenario, the zero VAT scenario would prevent 510 (0·2 %) IHD cases, 730 (0·4 %) stroke cases and 1570 (0·2 %) deaths. At the same time, the 20 % subsidy scenario would prevent 2010 (0·8 %) incident IHD cases, 2830 (1·7 %) stroke cases and 6130 (0·8 %) deaths. Under the assumption that the whole population would consume the recommended five portions of fruits and vegetables per day, as illustrated by the guideline scenario, the number of incident IHD and stroke cases would be reduced by 23 080 (9·0 %) and 30 510 (17·8 %), respectively, and there would be 67 560 (9·0 %) fewer deaths. Thereby, the prevented IHD, stroke and death cases under the zero VAT scenario correspond to 2·2–2·4 % of prevented cases under the guideline scenario, while the prevented IHD, stroke and death cases under the 20 % subsidy scenario correspond to 8·7–9·3 %.

### Ischaemic heart disease, stroke and death incidents cumulated over 10-year projection period

Under the reference scenario, the cumulative number of incidences would sum up to 2 766 070 IHD cases, 1 894 350 stroke cases and 8 506 500 deaths over the projection period of 10 years. The zero VAT scenario would prevent 4450 (0·2 %) of these incident IHD cases, 7010 (0·4 %) of these stroke cases and 13 960 (0·2 %) of these deaths. This translates to 2·1, 2·3 and 2·2 % of IHD, stroke and death cases that could be reduced under the guideline scenario, that is, if the whole population consumed the recommended five portions of fruits and vegetables per day. The 20 % subsidy scenario would prevent 17 990 (0·7 %) incident IHD cases, 27 390 (1·4 %) incident stroke cases and 54 880 (0·6 %) deaths, compared to the reference scenario. This translates to 8·5, 9·0 and 8·7 % of IHD, stroke and death cases that could maximally be prevented, that is, if the whole population consumed the recommended five portions of fruits and vegetables per day.

### Prevalent cases of ischaemic heart disease and stroke in projection year 10

In projection year 10, there would be 3 694 480 (4·6 %) prevalent IHD cases and 1 600 050 (2·0 %) prevalent stroke cases if fruit and vegetable intake remained unchanged. 1300 fewer prevalent IHD and 3410 fewer prevalent stroke cases could be expected under the zero VAT scenario, which represents 1·8 and 2·2 % of what could be achieved under the guideline scenario. Under the 20 % subsidy scenario, there would be an estimated 5780 and 13 680 fewer prevalent IHD and stroke cases, which represents 7·8 and 8·9 % of what could be achieved under the guideline scenario.

## Discussion

In order to quantify the prevention potential of fiscal policies targeting fruits and vegetables, we modelled health impacts following two fruit and vegetable subsidies, in comparison to an unchanged fruit and vegetable intake and a population-wide fruit and vegetable intake of five portions per day.

Our results show that the cumulated 4450 incident IHD cases, 7010 stroke cases and 13 960 deaths that would be averted under the zero VAT scenario over the 10-year projection period only prevent a fraction of the incidents that would occur under the reference scenario. Nonetheless, they translate to around 2 % of what could be maximally be reduced if the whole population consumed the recommended five portions of fruits and vegetables per day. Similarly, the 17 990 incident IHD cases, 27 390 stroke cases and 54 880 deaths cases that would be avoided under the 20 % subsidy scenario, cumulatively over 10 years, only prevent around one in a hundred incidents that would occur under the reference scenario. Nevertheless, this translates to around 9 % of what could maximally be reduced if the whole population consumed the recommended five portions of fruits and vegetables per day.

The number of prevented IHD, stroke and death cases over all scenarios is similar for males and females, except for the guideline scenario, which represents the health benefits that could maximally be achieved with any fruit and vegetable intervention.

DYNAMO-HIA is in established tool for health impact assessments^(27,32,35–37,40–43)^. In the following, we discuss the input data used for this health impact assessment.

Fruit and vegetable intake data were derived from GEDA 2012, even though a more recent wave (GEDA 2014/15-EHIS) is now available. This was decided because the more recent GEDA 2014/15-EHIS public use file only reports the consumed portions for people who stated they consume fruits and vegetables at least daily. In contrast, the GEDA 2012 public use file additionally reports the consumed portions for people who stated they consume fruits and vegetables less frequently, that is, once per week.

We used price elasticities specific to the German context^(28)^. These have previously been used in a similar study, which estimated the effect that removing the VAT on fruits and vegetables would have on obesity^(24)^. The price elasticities we used (–0·80 for fruits, –0·55 for vegetables) are similar to those presented in a systematic review for the US context (–0·70 for fruits, 0·58 for vegetables)^(44)^ as well as to those for the UK context (–0·70 for fruits, –0·63 for vegetables)^(45)^.

It was not possible to consider cross-price elasticities in this model because the DYNAMO-HIA tool only allows for the use of one risk factor at a time. Cross-price elasticities indicate that a 10 % price reduction for fruits would increase the demand for meat by 0·3 %, for vegetables by 1·3 %, for cereals by 0·8 %, and decrease the demand for potatoes/rice/noodles by 0·4 %. A 10 % price reduction for vegetables would increase the demand for meat by 0·7 %, for fruits by 0·9 %, for potatoes/rice/noodles by 0·4 %, for cereals by 0·6 %, and decrease the demand for milk products by 0·1 %^(28)^. The inclusion of cross-price elasticities is therefore unlikely to change our results.

There is possible evidence that fruit and vegetable intake prevents body weight gain^(1)^ and reduces overweight and obesity^(46)^. Further, there is convincing evidence that fruit and vegetable intake has a BP-lowering effect^(1)^ and reduces the risk for hypertension^(47)^. Even though these risk factors for IHD and stroke were not separately incorporated into our simulation, it can be expected that the VAT removal on fruits and vegetables would simultaneously contribute to lower levels of obesity and hypertension.

Finally, relative risks for cancer were not included in our simulation. Previous literature has shown that increasing the intake of fruits and vegetables to the recommended level of 500 g/d in France, Germany, the Netherlands, Spain and Sweden would prevent 398 out of 211 708 fruit- and vegetable-related cancer cases in 2050^(48)^. Therefore, we can assume that the VAT removal on fruits and vegetables would have additional health benefits due to prevented cancer cases.

## Conclusion

Fiscal policies on fruits and vegetables provide a non-negligible step towards the removal of the health burden induced by low fruit and vegetable intake.
